# The FitSpirit approach for increasing physical activity in canadian teenage girls: protocol of a longitudinal, quasi-experimental study

**DOI:** 10.1186/s12889-021-10200-5

**Published:** 2021-01-28

**Authors:** Geneviève Leduc, Jo-Anne Gilbert, Alexandra Ayotte, Nicolas Moreau, Vicky Drapeau, Jean Lemoyne, Johana Monthuy-Blanc, Jonathan Tremblay, Marie-Eve Mathieu

**Affiliations:** 1 FitSpirit, 141, rue Saint-Charles, Bureau 4, Sainte-Thérèse, Quebec J7E 2A9 Canada; 2 École de kinésiologie et des sciences de l’activité physique, Université de Montréal, P.O. Box 6128, Downtown Station, Montreal, Quebec H3C 3J7 Canada; 3 School of Social Work, University of Ottawa, 120 University Private, Ottawa, Ontario K1N 6N5 Canada; 4 Département d’éducation physique, Université Laval, 2300 Rue de la Terrasse, Quebec City, Quebec G1V 0A6 Canada; 5 Département des sciences de l’activité physique, Université du Québec à Trois-Rivières, 3351, boul. des Forges, Trois-Rivières, Quebec G9A 5H7 Canada; 6 Sainte-Justine University Health Center, 3175 Chemin de la Côte-Sainte-Catherine, Montreal, Quebec H3T 1C5 Canada

**Keywords:** Adolescent girls, Extra-curricular intervention, Health, Lifestyle, Physical activity, Quantitative methods, School-based program

## Abstract

**Background:**

Worldwide, most adolescent girls do not meet physical activity (PA) recommendations and very few PA programs are tailored specifically towards them. Even fewer information exists about the long-term effects of such programs. Some Canadian schools have implemented the FitSpirit PA intervention designed specifically for girls aged 12 to 17 years old. This paper describes the protocol of a quasi-experimental study evaluating long-term changes in health behaviours and outcomes following FitSpirit participation.

**Methods:**

The study is conducted among schools that partner with FitSpirit every year. It started in 2018 and will be completed in 2022. The intervention comprises motivational talks, a turnkey running program, PA sessions and special events. Study participants fill out an online questionnaire twice a year. Follow-up questionnaires are sent at the end of each school year to the study participants who dropout from FitSpirit. The main outcome, changes in PA levels, is evaluated using questions validated for adolescents. Secondary outcomes are health (perceived health); lifestyle habits (sedentary activities, eating and sleeping habits); psychosocial outcomes (physical self-efficacy and body satisfaction); and FitSpirit appreciation (activity participation and satisfaction). Most questions originate from questionnaires validated for the adolescent population. Cross-sectional and longitudinal analyses will be performed.

**Discussion:**

This study will provide one of the first longitudinal reports on the impact of a large extra-curricular PA intervention designed specifically for adolescent girls. The current study will uniquely contribute to PA research by assessing outcomes additional to PA levels, including markers of health, lifestyle habits and psychosocial determinants.

**Trial registration:**

NCT, NCT03804151, Registered on January 22, 2019; retrospectively registered.

**Supplementary Information:**

The online version contains supplementary material available at 10.1186/s12889-021-10200-5.

## Background

Physical inactivity has major deleterious effects on the physical, mental, psychosocial and emotional health of adolescents [1]. Systematic reviews, meta-analysis and original research support that the promotion of an active lifestyle at this stage of life is essential because it contributes to better health outcomes during adolescence [2, 3] and prevents the development of chronic diseases and health disorders later in life [4, 5]. Unfortunately, very few adolescents take advantage of the health benefits of being physically active. Worldwide estimates reveal that only 1 in 4 adolescents meet the recommendation of 60 min of moderate-to-vigorous physical activity (PA) daily [6]. Recent data among canadian adolescents resulted in very similar statistics with 24% not meeting the recommendations and girls being less active than boys (14% vs. 34%, respectively) [7]. Systematic reviews and original research support that the decline in PA levels can be first observed in childhood, even though girls tend to disengage from active pursuits at a steeper rate as they reach puberty and progress through adolescence [8, 9].

In response, interventions have been put in place to promote the achievement of PA guidelines among adolescent girls specifically. Schools have been identified as an ideal setting to promote healthy lifestyles for adolescents, since the social environment, and more specifically their peers, can influence them greatly [10]. In addition, recent systematic reviews suggest that school-based PA interventions are the most promising avenues to increase adolescent girls’ PA levels [11, 12]. A meta-analysis highlights that there is also evidence that girl-only interventions can lead to a greater impact on PA levels than mixed-sex interventions [12].

Nevertheless, a limited but growing number of school-based girl-only PA interventions have been reported [13–18]. In addition, studies that evaluated school-based interventions for adolescent girls on PA levels generally have a small sample size [13], with only two known studies that used a sample larger than 1000 adolescent girls [18–20]. In the literature, there are also very few girl-only extra-curricular interventions [13]. Most meta-analysis and studies on PA for girls solely evaluated the intervention’s effect on PA levels [11–13, 21, 22], neglecting to assess global outcomes of interest, such as health, lifestyle including sedentariness and psychosocial markers. There is some preliminary evidence that PA programs could improve sleep quality of adolescents, but no analyses have been performed by sex [23]. However, there is some evidence that girls-only PA interventions could yield improvements in eating habits [24] and different fitness markers [25, 26]. Furthermore, our recent systematic literature search showed that evidence on the physiological and psychological effects of PA programs designed for adolescent girls is limited and comes from studies of moderate methodological quality [27]. Interestingly, very few of these studies evaluated self-efficacy, though there is a growing interest on the topic [18]. In addition, long-term assessments of participants has been highlighted as a current limit in this field of research [27].

Evidence to date highlights the need for additional longitudinal studies, with a large sample size, conducted in real-life settings to draw clearer conclusions on the effects of school-based PA interventions specifically tailored to adolescent girls. Real life studies inform on the effectiveness of interventions implemented in routine circumstances [28]. FitSpirit is a non-for-profit organization that has been offering school-based, girl-only PA program in Canada since 2007. Their intervention is promising due to the non-competitive and mobilization approaches but remained understudied. In 2017–2018, over 12,000 girls participated regularly in FitSpirit activities delivered in 250 partner schools. Through their activities and event offerings, FitSpirit aims to get adolescent girls moving, and keep them physically active over the long term. The objective of this article is to describe the protocol used to evaluate the changes resulting from the implementation of the FitSpirit approach in schools. It presents a longitudinal, quasi-experimental, non-controlled study that aims to evaluate the following outcomes:

Primary outcome: Changes in PA levels among teenage girls who take part in FitSpirit activities in their school.

Secondary outcomes: Health (perceived health); lifestyle habits (sedentary activities, eating and sleeping habits); and psychosocial determinants (physical self-efficacy and body satisfaction) associated with the FitSpirit program. The study also aims to evaluate the girls’ level of participation as well as their appreciation towards some aspects of the FitSpirit approach.

## Methods

The present article has been written following the guidance of the Standard Protocol Items: Recommendations for Interventional Trials (SPIRIT) [29], as suggested [30]. See Additional file 1 for more details.

### Design of the FitSpirit approach

FitSpirit partners with schools to provide activities that get young girls moving and keep them physically active over the long term. It offers tools and services within a holistic approach based on flexible, individualized support to help schools engage girls through girl-only activities tailored to their challenges and their preferences. Non-competitive group activities are planned, organized and led by a member of the school staff, the school program leader, with the support from a regional coordinator. The physical and health education teacher, or any other school employee, can become the FitSpirit school leader. FitSpirit activities can run during the entire school year, with most schools organizing activities only between February and May and for a minimum of 8 weeks (unpublished data). For example, only 67 of the 285 partner schools registered in the school year 2018–2019 have organized FitSpirit activities during fall. In addition, during this same school year, an average of 19 activities have been organized by the partner schools.

FitSpirit offers its support through several services, but mainly the ones listed below. Each partner school decides the extent to which they use and exploit these every year and this information is collected by FitSprit in order to conduct a process evaluation.

- FitSpirit Ambassadors: Ambassadors are dynamic, passionate women who inspire girls and motivate them to be physically active. They act as accessible role models while representing the organization in diverse settings. Their message is conveyed through motivational conferences delivered in school. They also lead various PA sessions as a way to introduce girls to different ways of being active, such as yoga classes, zumba, bootcamp, rugby, orienteering, etc. The school leaders choose which activity is best for participants. Every year, more than 150 women take on this role for FitSpirit.
- An interactive web platform that includes a dynamic tool for choosing workouts, tips for being active, videos, recipes, and the service of a nutritionist and a kinesiologist to answer questions.
- An 8–10-week turnkey running program: The program suggests an 8-week exercise progression including strength and intervals trainings. It has been developed for adolescents’ girls. Exercise intensity is determined using a perceived exertion scale.
- Major events: Yearly, FitSpirit holds a series of events that bring together thousands of girls for a memorable, high-energy day of PA and togetherness. Participants can tackle a range of exciting challenges, from completing a not-timed 5 K or 10 K run, to trying out any number of fun new activities (e.g. baseball, obstacle course, judo, ultimate Frisbee). Every girl, regardless of skill or fitness level, is invited to pursue her discovery of sports, PA and the great outdoors.
- FitSpirit training: Yearly, in-person training sessions aimed at program leaders and ambassadors are delivered in several locations. Webinars and online tools are also available to those who are unable to attend. The training provides: 1) Hands-on tools for the FitSpirit community to help them communicate the joy and benefits of adopting a healthy lifestyle; 2) Leadership and outreach training for Student Leaders; 3) Continuing education courses and tools specially tailored to the priorities and challenges identified by FitSpirit; 4) Spaces for the FitSpirit community to share knowledge and best practices.

Readers are invited to visit the website (www.fitspirit.ca) for further information about the FitSpirit approach.

### Study population

All Canadian schools with girls 12–17 year old are invited to implement the FitSpirit approach. Yearly, between 250 and 300 schools from the provinces of Quebec and Ontario become FitSpirit partners. School boards, principals and teachers learn about the organization through advertisement, word-of-mouth and their regional coordinator. A regional coordinator acts as an advisor, a resource person and an advocate for PA in the community and is the FitSpirit liaison for the field team in each geographic area. This person contributes to the promotion and deployment of the approach in non-partner schools. It is up to each school to decide whether all or only a limited group of the female student population will participate in the activities and events planned by the school. Although the approach aims to encourage inactive girls to participate, this is not a participation criterion and therefore all are welcomed. Therefore, FitSpirit participants have mixed sport backgrounds and abilities.

**Fig. 1:**
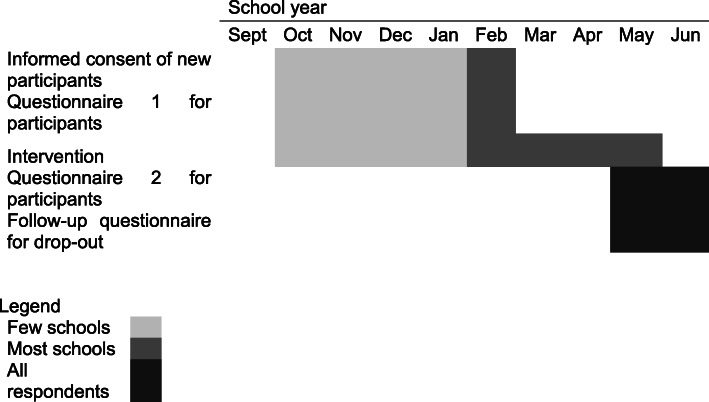
Schedule of enrolment, intervention, and assessments within a school year

The FitSprit registration process includes an invite to provide consent for the participation of the girls in the research project. As required by the Quebec law, participants younger than 14 have to provide one parent’s written informed consent and those aged 14 years and older can provide their own written consent. Information and consent form is available online (fillactive.com). The FitSpirit organization collects research consent information because they transfer only anonymous data to the research team. Figure 1 shows the timeline of the data collections within each school year.

**Fig. 2:**
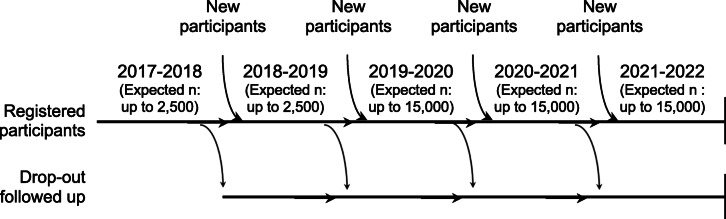
Schedule of enrolment, intervention, and assessments over the course of the project

- Between 2017 and 2019, a stratified random sampling method was applied to recruit girls for research. Each school leader randomly selected five to ten research participants among all girls who provided consent, irrespective of grade. The FitSpirit organization sent the invitation to research participants via email and their school leader could help them have access to the online questionnaires at school.
- By the year 2019–2020, the FitSpirit organization moved towards the invitation of all girls with consent to participate in the research project (15,000 potential participants). FitSpirit send invitations for the research via email and the school leaders are no longer involved in research.
- For the follow-ups, the same girls are contacted again by the FitSpirit organization, whether or not they still participate in FitSpirit in subsequent years, as illustrated in Fig. 2.
- Research participants will be contacted for follow-ups each year until May 2022.

### Data collection

Data are collected by the FitSpirit organization using an online questionnaire (Survey Monkey) available in both French and English. Table 1 presents the variables collected as well as the source of the questions. Most questions come from the Canadian Health Measure Surveys [31, 35] and the *Enquête Québécoise sur la santé des jeunes du secondaire* [32], two large surveys that have been validated for a Canadian teenage population. Moreover, a validation phase for the questionnaire was run among a small group of FitSpirit participants in 2017 where additional in-house questions were tested. Based on the results and the experience gained during this phase, a group of seven co-investigators with complementary expertise in exercise, nutrition, social and health sciences developed the content of the current questionnaire. Many rounds of consultations led to the final content and question order. Table 2 presents the questions used to evaluate PA levels of participant, that is the primary outcome of the study. The Additional file 2 shows the questionnaire to be answered on Survey Monkey by participants at the end of a school year.

**Table 1:** Variables measured in the questionnaire and question’s source

Category	Variable
General information	• Age (in-house questionnaire) • School (in-house questionnaire) • School grade (in-house questionnaire) • Postal code (in-house questionnaire)
PA levels (primary outcome)	• Presence of PA facilities in community (in-house questionnaire) • Days per week with 60 min of PA [31] • Time per week using active transportation modes [31] • Time per week in leisure time PA [31] • Group vs. individual PA (in-house questionnaire) • School vs. outside school PA (in-house questionnaire)
Health (secondary outcomes)	• Perceived health status [31] • Weight [32] • Height [32] • Physical or mental health conditions diagnosed [31]
Lifestyle habits (secondary outcomes)	• Food consumption habits [32] • Breakfast eating [32] • Sleep time and somnolence [31] • Screen time per week [31]
Psychosocial outcomes (secondary outcomes)	• Perceived and desired body image [32] • Weight control techniques [32] • Physical self-efficacy [33] • Quality of life [31] • Physical self-esteem [34] • Interest in school [32] • Sense of belonging [35]
FitSpirit appreciation (secondary outcomes)	• Reasons for participation (in-house questionnaire) • Words associated with FitSpirit (in-house questionnaire) • Participation level (in-house questionnaire) • Satisfaction level (in-house questionnaire) • Injuries (in-house questionnaire)

*PA* Physical activity

**Table 2:** Questions evaluating physical activity levels

Questions	
• Over a typical or usual week, on how many days are you physically active for a total of at least 60 min in total per day? Consider only the activities that made you breathe harder and sweat at least a little. ○ None (zero days) ○ 1 day ○ 2 or 3 days ○ 4 to 6 days ○ Everyday	
• In the last 7 days, how long did you use active forms of transportation to get around, like walking to school or cycling to get to work, the shopping center or a friend’s place? (Enter hours AND minutes. You can enter 0 h and 0 min if you have not used active modes of transportation.)	
• In the last 7 days, how long did you do physical activity in your leisure time including exercising, playing an organized or non-organized sport or playing with your friends? (Enter hours AND minutes. You can enter 0 h and 0 min if you have not been active during your leisure time.)	

Data are collected at the beginning and at the end of each school year. Study participants are invited via their personal email address provided at registration to complete the online questionnaire. The end of year questionnaire is also sent to study participants who dropped out from FitSpirit. The longitudinal design of the study will allow the research group to perform an assessment over a prolonged period of time. It will also allow for an evaluation of the persistence of the effects of the program over the summer school break.

To ensure participant retention at each data collection period, research participants can enter a draw to win prices. Prices for a total value per year of approximately $1000 are given among participants who fill out the online questionnaire. There is a new draw at each data collection period and participants have one chance to win per period. Data integrity is enforced using different mechanisms. First, participants complete themselves the online questionnaire and answers will be transferred directly into the database. This procedure eliminates the risk of transcription errors. Then, verification for valid values and consistency against data already stored in the database will be performed. The answers to the questionnaires will remain stored online and a password system will be utilized to control access. Databases will also be secured with password-protected access systems.

### Sample size determination

This protocol presents the first attempt to evaluate the overall effect of the FitSpirit approach. The primary outcome of this project is PA level. For our specific population, there is no direct comparative reference that can be used to perform a relevant power calculation. Most studies used accelerometer data to evaluate the impact of their intervention on PA levels and authors used different questions [13]. Since the FitSpirit intervention is applied in many schools covering a large territory, a great number of participants is required to be representative of the various environments where the intervention is used. The authors have thus decided with the organisation to include all participants, leading to a potential sample of 15,000 individuals. For logistical reasons, it is unrealistic to measure objectively the PA levels of all participants. To our knowledge, the largest study to evaluate self-reported PA levels among adolescent girls in response to a school-based intervention included approximately 3000 participants [36]. They observed a significant 8% increase in the proportion of girls who reported engaging in vigorous PA, that is activities assigned at least 6 metabolic equivalents (METs). It is then reasonable to think that 15,000 participants will lead to enough power to detect meaningful changes in PA levels.

### Statistical analyses

The primary research question to be answered relates to the impact of FitSpirit on the participants’ PA level. The secondary research questions relate to the impact of FitSpirit on other lifestyle habits (sedentary activities, eating and sleeping habits), health perception, physical self-efficacy, body satisfaction, and participants’ appreciation of the FitSpirit approach. Both continuous (ex: total minutes of PA/week) and categorical data (ex: meeting or not the recommendation for moderate-to-vigorous PA) will be used as dependent variables, while the number of FitSpirit activities/year and of years of participation at FitSpirit will be used as independent variables. Data will be analysed in a cross-sectional fashion using chi-square test and Fisher’s exact test (depending on the sample size) to evaluate for instance the relationship between categories of PA levels and categories by the number of years of participation in FitSpirit. Pearson’s test will assess, for example, the correlation between the number of days/week meeting PA recommendations and levels of physical self-efficacy. In addition, analyses of variances will compare the levels of PA (total minutes of PA/week) between grade levels. Longitudinal analyses will evaluate primarily the change in PA levels, and in other secondary outcomes afterwards. Statistical adjustments will be performed using, for instance, the variation in the length of intervention from one school to another or the baseline lifestyle habits of participants as covariates.

## Discussion

This study will make a unique contribution to the research literature by providing one of the first longitudinal assessments of a large extracurricular PA intervention designed specifically for adolescent girls, satisfying the important knowledge gap concerning large-scale evaluations of girl-only interventions. It will document its impact on PA levels, which is of crucial importance considering the disengagement from PA participation among this population. It will also be the first study to document the impact on other outcomes such as health, lifestyle and psychosocial indicators (Table 1). To our knowledge, this study will be the first to document the impact of a girl-only PA intervention on perceived body image among girls. This will certainly help better understand the global impact of such interventions. Results will also contribute to increasing the quality and effectiveness of the FitSpirit approach and other girl-only PA interventions. Even though it is not the focus of this intervention, we will also document the impact of such an innovative girl-only approach on obesity prevention.

This study presents many strengths. First, the protocol development process included a pilot study. In addition, most of the questions in the questionnaire have been validated for the specific population assessed (Table 1). Second, this quasi-experimental longitudinal study collects data in real-life school-settings where leaders are supported through a flexible approach in which they can select various FitSpirit activities that best fit their specific needs and challenges. The high number of participants to be recruited is another strength. Data are collected at the beginning and at the end of each school year, a strategic positioning given that the intervention is school-based. Furthermore, the collaborative work of the co-investigators that come from different backgrounds and include various expertise allows this study to assess a broad range of outcomes. The quasi-experimental design of this study comes with some limitations, such as the absence of a control group. In this regard, the co-investigators consider the possibility to compare the results with those collected by large national and provincial surveys, especially since national and provincial questionnaires are used with FitSpirit. The use of self-reported measures could also lead to some biases, such as the social desirability bias. Participation bias cannot be excluded also. One could think that only the most motivated participants would fill the questionnaires. In addition, because each school decides who participate in the FitSpirit activities, it cannot be excluded that in some rare circumstances some schools allow only a specific subgroup of girls to join the program and this subgroup may not represent the general population of adolescent girls.

By documenting the global impact of a holistic school-based girl-only intervention, this study could improve the quality and effectiveness of interventions aimed at promoting PA among girls.

### Trial status

This article presents the protocol as last approved on May 15, 2019. The recruitment began on February 2018 and will be completed at the end of 2022.

## Supplementary Information

**Additional file 1.** SPIRIT checklist.

**Additional file 2.** End of school year questionnaire used to evaluate the FitSpirit approach

## Data Availability

Data sharing is limited to the study research team and all analyses will be carried out internally. Each paper or abstract must be submitted to the Subcommittee for review of its appropriateness and scientific merit prior to submission. The Subcommittee may recommend changes to the authors.

## Abbreviation

PA: physical activity
