# Supplementary figures and images for: The FitSpirit approach for increasing physical activity in canadian teenage girls: protocol of a longitudinal, quasi-experimental study
Source: BMC Public Health. 2021 Jan 28;21:229. doi: 10.1186/s12889-021-10200-5 (PMC7841897; doi:10.1186/s12889-021-10200-5)

**
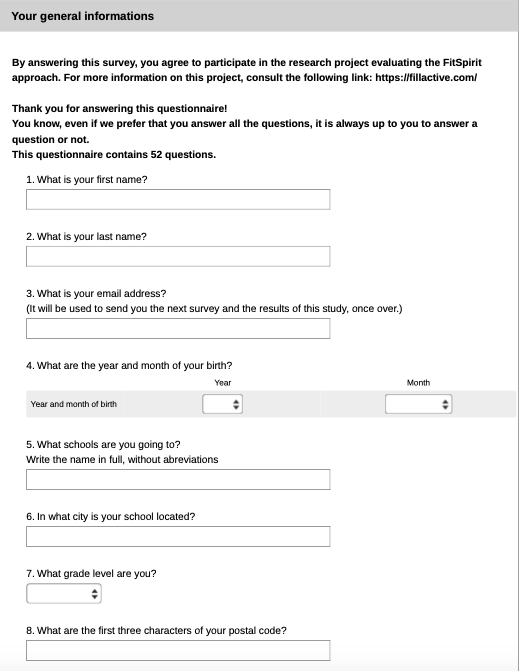
**

**
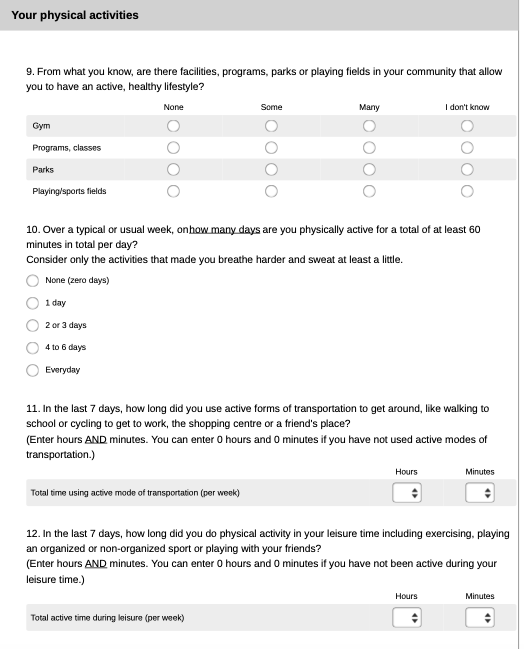
**

**
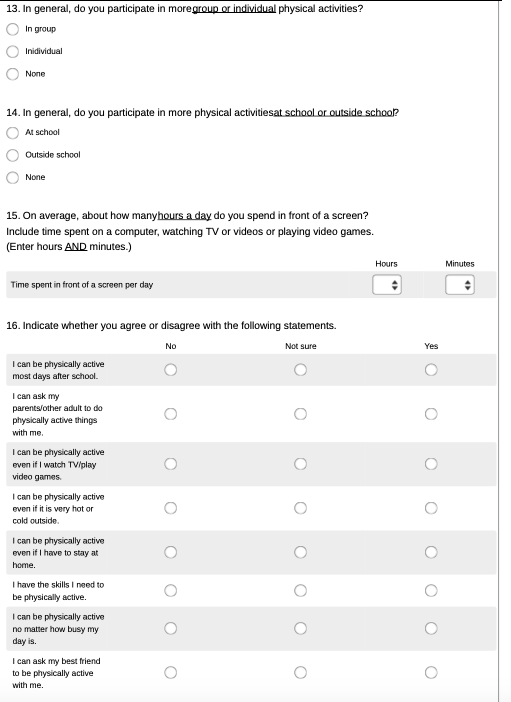

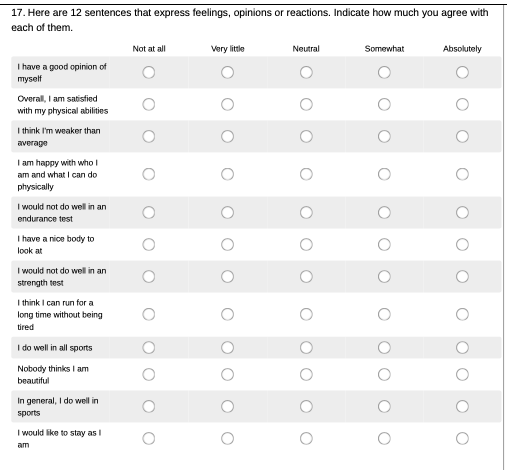

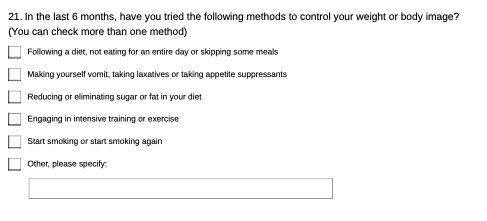

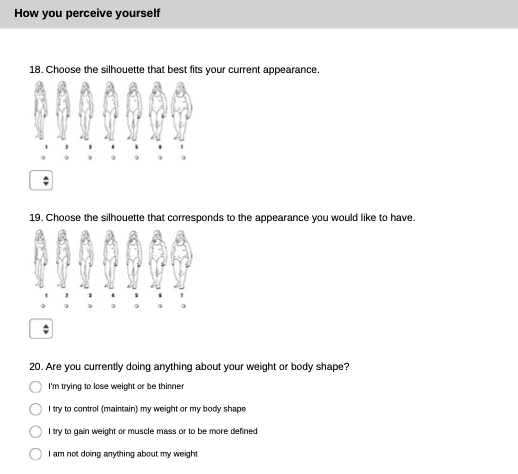

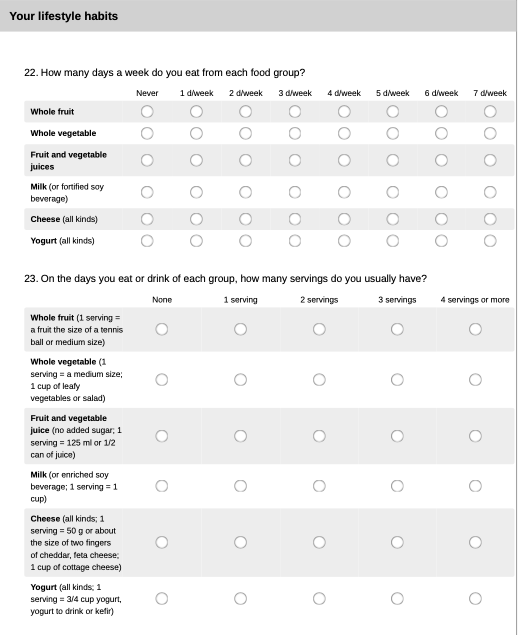

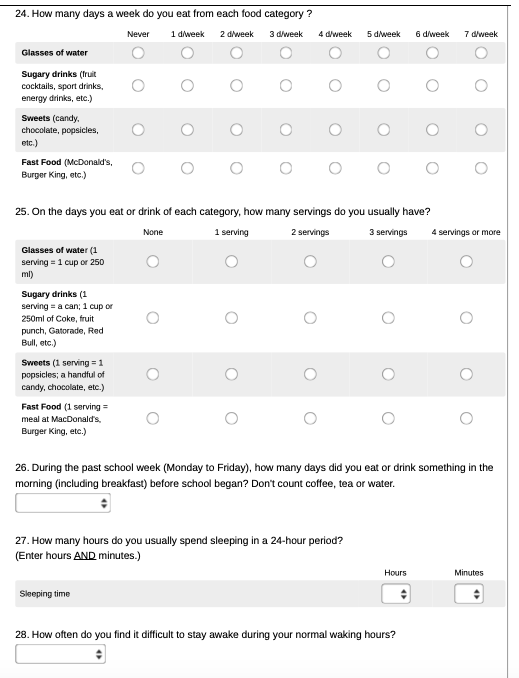

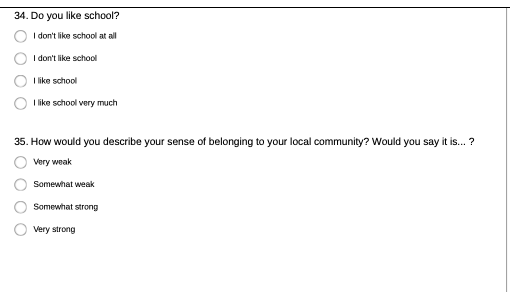

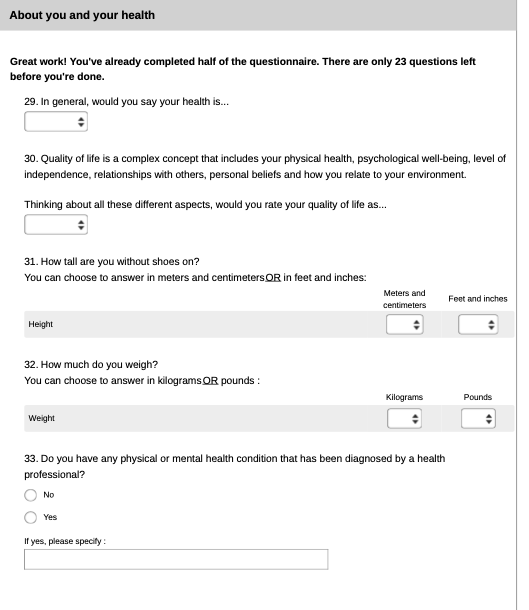

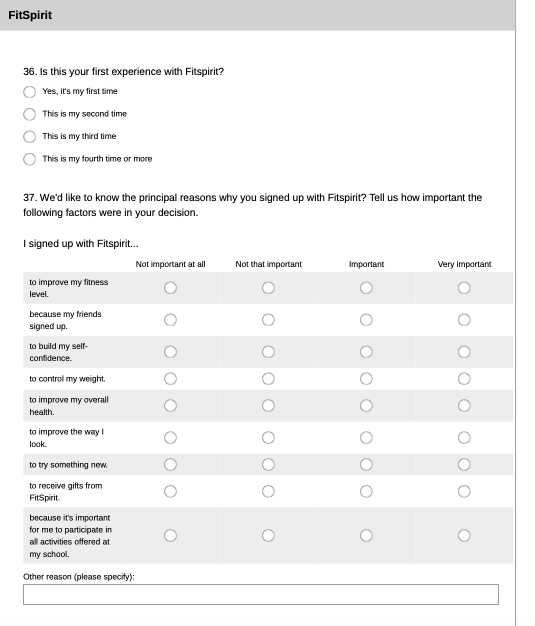

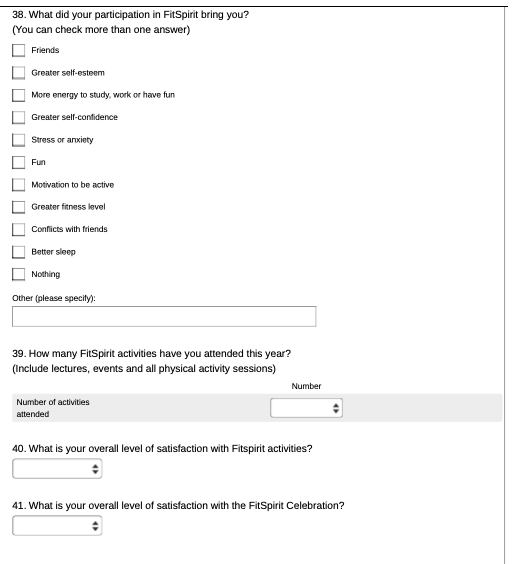

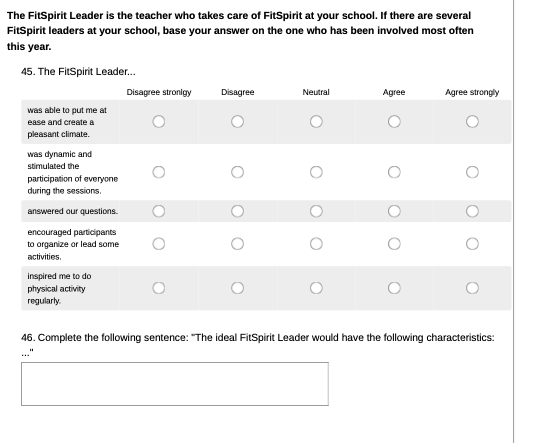

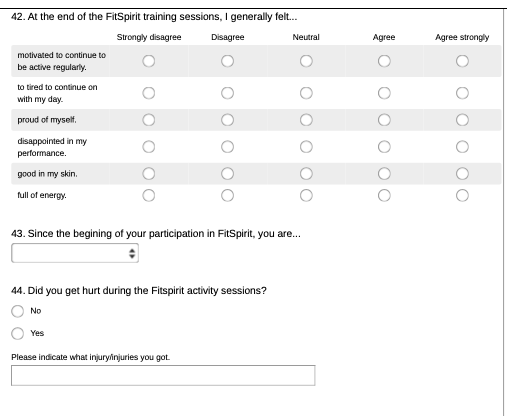

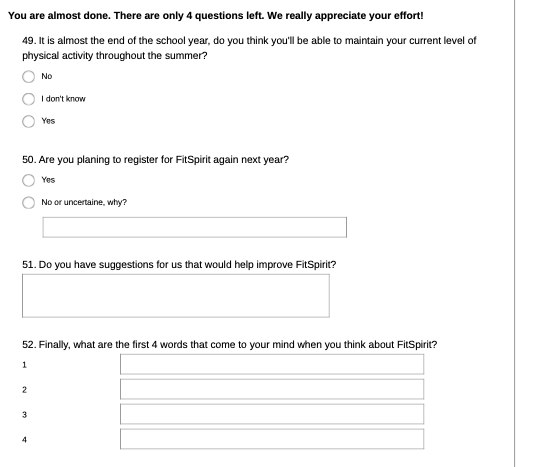

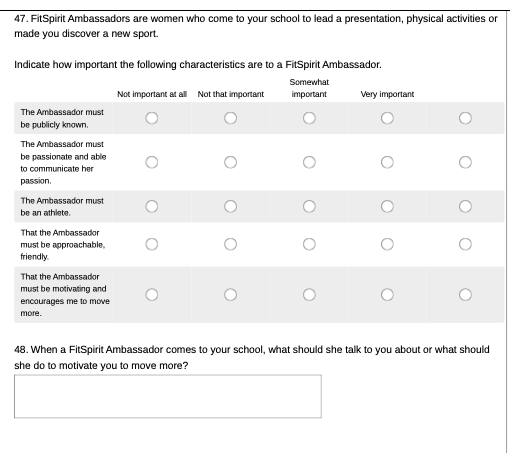
**

Supplement: Supplementary file 2 — Additional file 2. End of school year questionnaire used to evaluate the FitSpirit approach [file 12889_2021_10200_MOESM2_ESM.docx]
